# The Value of Preoperative Volumetric Analysis by Computerised Tomography of Retrosternal Goiter to Predict the Need for an Extra-Cervical Approach

**DOI:** 10.4274/balkanmedj.2017.0161

**Published:** 2018-01-20

**Authors:** İsmail Cem Sormaz, Derya S. Uymaz, Ahmet Y. İşcan, İlker Özgür, Artur Salmaslıoğlu, Fatih Tunca, Yasemin G. Şenyürek, Tarık Terzioğlu

**Affiliations:** 1 Department of General Surgery, İstanbul University İstanbul School of Medicine, İstanbul, Turkey; 2 Department of General Surgery, Koç University School of Medicine, İstanbul, Turkey; 3 Clinic of General Surgery, Acıbadem International Hospital, İstanbul, Turkey; 4 Department of Radiology, İstanbul University İstanbul School of Medicine, İstanbul, Turkey; 5 Clinic of General Surgery, Amerikan Hospital, İstanbul, Turkey

**Keywords:** Sternotomy, thyroidectomy, surgery, thyroid, computerized tomography, retrosternal goiter, substernal

## Abstract

**Background:**

A thyroidectomy can be performed via a cervical incision in most patients with retrosternal goiter.

**Aims:**

To investigate the correlation between the volume of the mediastinal portion of the thyroid gland and the need for an extra-cervical approach for retrosternal goiter.

**Study Design:**

Diagnostic accuracy study.

**Methods:**

The measurement of craniocaudal length and the volume of the mediastinal component of the thyroid gland on computerised tomography images was performed in 47 patients with retrosternal goiter. Of these 47 patients, 8 (17%) required an extra-cervical approach and were classified as group 1, and 39 (83%) patients that required a cervical incision were classified as group 2. Receiver operating characteristic analysis was performed to determine the cut-off value for the craniocaudal length and the volume of the mediastinal thyroid mass, which significantly correlated with an extra-cervical approach for retrosternal goiter.

**Results:**

Reoperative surgery was significantly more frequent in group 1 than in group 2 (50% vs 13%; p=0.03). The craniocaudal length of the mediastinal thyroid gland was significantly longer in group 1 than in group 2 (77±11 mm vs 31±21 mm, respectively; p=0.0001). The volume of the mediastinal component was significantly larger in group 1 compared to group 2 (264±106 cm^3^ vs 40±41 cm^3^, respectively; p=0.0001). The receiver operating characteristic curve of craniocaudal length and the volume of the mediastinal component identified ≥66 mm and ≥162 cm^3^ as the cut-off values with the maximum accuracy, respectively. The craniocaudal length of the thyroid mass below the thoracic inlet ≥66 mm or a volume of the mediastinal portion ≥162 cm^3^ were significantly associated with an extra-cervical approach (p=0.0001). For predicting an extra-cervical approach, the sensitivity, positive predictive value and negative predictive value of the cut-off value for craniocaudal length was 87.5%, 64% and 97%, respectively. For predicting an extra-cervical approach, the sensitivity, positive predictive value and negative predictive value of the cut-off values for the mediastinal volume were 100%, 89% and 100%, respectively.

**Conclusion:**

A thyroid volume of ≥162 cm^3^ extending below the thoracic inlet was a significant determining factor for an extra-cervical approach, with a negative predictive value for the extra-cervical approach of 100% for retrosternal goiter with smaller volumes. Further studies with an increased number of patients are needed to determine the value of volumetric analysis of retrosternal goiter to predict the need for an extra-cervical approach in retrosternal goiter.

The incidence of the substernal or mediastinal extension of a goiter ranges between 2.6% and 30.4% (1,2,3,4,5,6). Lack of consensus about the definition of a retrosternal goiter (RSG) or the use of different criteria for the selection of patients in a published series may explain this extensive difference. The most commonly accepted definitions used to describe an RSG are a goiter that descends below the plane of the thoracic inlet or a goiter that has more than 50% of its mass lying inferior to the thoracic inlet (7,8,9). Because a large portion of the thyroid gland is located in the chest, the thyroid gland of 20% to 30% of patients with RSG cannot be palpated during neck examination (7,8). Computerised tomography (CT) is the most widely used technique to visualise RSG, which enables the assessment of morphology, size, the site of mediastinal extension and the relation to the adjacent mediastinal structures, vessels, and organs (10,11). The treatment for RSG is surgical unless the patient has serious co-morbidities that pose a high operative risk (2,7,9,12). A thyroidectomy can be performed via a cervical incision in most patients with RSG, and the reported percentage of extra-cervical approach usually does not exceed 10% (12,13). Previous thyroid surgery, posterior mediastinal extension, a primary substernal goiter, a dumbbell-shaped goiter, invasive thyroid cancer and the extension of RSG to the level of the aortic arch or carina are the most prominent factors that lead to an extra-cervical approach (10,12,13,14,15). Several classification systems that mainly depend on CT findings were defined for the preoperative stratification of patients with RSG who would require an extra-cervical approach (10,11,12,13,14,15). Most of these systems were based on the craniocaudal (CC) length of the mediastinal extension, anterior or posterior mediastinal extension and the relationship of the substernal thyroid gland to major vessels and trachea. To the best of our knowledge, there is no study in the literature that has evaluated the impact of the volume of the mediastinal portion of an RSG on the need for an extra-cervical approach.

This study is to investigate the correlation between the volume of the mediastinal portion of the thyroid gland and the need for an extra-cervical approach in patients with RSG.

## MATERIALS AND METHODS

Two hundred and four patients underwent a thyroidectomy for RSG between January 1990 and January 2016 in our clinic. An extra-cervical approach was needed in 20 (9.8%) patients. In our institution, the accessibility to multi-slice CT images of RSG patients from an image database system was available after January 2014. This study included 47 (23%) of the 204 RSG patients who underwent a thyroidectomy between January 2014 and January 2016 and in whom volumetric measurement of the mediastinal thyroid gland could be accomplished from CT images obtained from the image database system. The mean age of these 47 patients was 57.3±11 years with a female/male ratio of 25/22. Of the 47 patients, 24 (51%) were symptomatic and 9 (19%) had previous thyroid surgery. The symptoms associated with RSG were compressive symptoms, such as dyspnea (n=19), dysphagia (n=12) and hoarseness (n=5). Seven patients complained of retrosternal pain. Eight (17%) patients had hyperthyroidism and were treated with antithyroid agents preoperatively. All patients underwent preoperative indirect laryngoscopy to evaluate vocal cord motility. Preoperative unilateral vocal cord dysfunction was found in 1 patient.

Physical examination of these 47 patients revealed that the lower portion of the thyroid gland remained permanently below the sternal notch with the neck in either the normal position or hyperextension. Neck ultrasound showed evidence of retrosternal extension of the thyroid gland in all patients. Therefore, all of the 47 patients underwent preoperative CT of the neck and thorax to reveal the extent of retrosternal extension. In our study, RSG was defined as clinical and radiological evidence of a thyroid gland descending below the plane of the thoracic inlet in which some portion remains permanently retrosternal. All of the surgical procedures were performed by the same surgical team, who are experienced in endocrine surgery. During the thyroidectomy, all cases were initially approached using a cervical incision. Initially, thyroid vessels were individually divided. After the liberation of the upper pole of the thyroid lobe, the thoracic component of the RSG was manually retracted to the cervical region with gentle digital blunt dissection. The extra-cervical approach was selected in cases in which the cervical approach was inadequate to provide optimal surgical access and blind dissection was considered unsafe in the aspect of the risk of recurrent laryngeal nerve damage or mediastinal hemorrhages or when a sizable mediastinal thyroid gland could not be extracted from the thoracic inlet. Patients who required an extra-cervical approach were classified as group 1 (n=8, 17%), and patients who required a cervical incision were classified as group 2 (n=39, 83%). Age, sex, previous thyroid surgery, the rate of compressive symptoms and hyperthyroidism, volume and CC length of the mediastinal portion of the thyroid gland were compared between the 2 groups. Written informed consent was obtained from each patient prior to surgery.

The Statistical Package for Social Sciences (SPSS) for Windows Version 21.0 (Armonk, NY: IBM Corp.) was used for statistical analysis. The continuous variables are summarised as mean ± standard deviation. The differences between groups were assessed using the Student’s t-test and a Mann-Whitney U test for normally distributed and non-normally distributed data, respectively. Fisher’s exact test was used to test differences in proportions. A p value <0.05 was considered to be statistically significant. Receiver operating characteristic (ROC) analysis was performed to determine the cut-off value for CC length and volume of the mediastinal thyroid mass, which significantly correlated with an extra-cervical approach for RSG. Ethics Committee approval was obtained (2017/657).

**Figure 1a, b:**
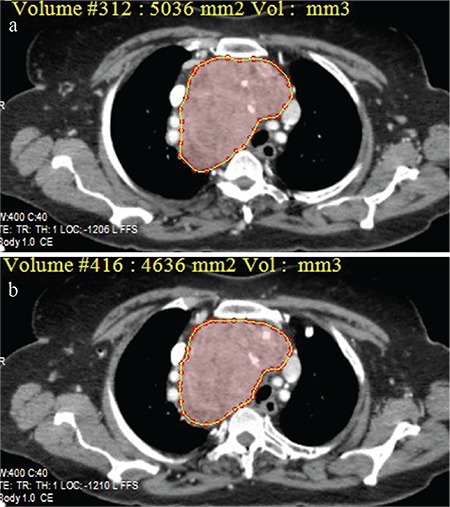
Estimation of the volume of the mediastinal thyroid mass by free-hand outlining of the perimeter of the mediastinal thyroid gland throughout the contiguous slices on multi-slice computerised tomography.

**Volumetric calculation: **Multi-slice CT (Toshiba Multi-slice Aquilion 64 system, Toshiba Medical Systems, Tokyo, Japan) was performed with 64x1 mm collimation and a rotation time of 0.4 seconds. The tube current was 300 mA at 120 kV. In obese patients (body mass index ≥30 kg/m2), the parameters were adjusted to 350 mA at 135 kV to improve image quality. The CT scan was performed with the patient in the supine position. The thyroid volume was measured through the contiguous slices below the thoracic inlet. The software enabled free-hand outlining of the perimeter of the mediastinal thyroid gland. All outlines were performed by 2 investigators trained to recognise the relevant organ boundaries (Figure 1a, 1b). The volume of the outlined thyroid gland was determined for each slice by a volume-rendering tool. The sum of the volume calculations of contiguous slices was divided by the slice number to estimate the thyroid volume.

## RESULTS

The rate of an extra-cervical approach was 17% (n=8). Of the 8 patients who required an extra-cervical approach, a sternotomy was performed in 7 patients, and a thoracotomy was necessary for 1 patient. The demographic data, the rates of hyperthyroidism, compressive symptoms, previous thyroid surgery, thyroid malignancy and the features of the RSG in groups 1 and 2 are summarised in Table 1. Age and sex showed no significant difference between the 2 groups. Although the rates of hyperthyroidism, compressive symptoms and posterior mediastinal extension were higher in group 1 than in group 2, the difference between the 2 groups was not statistically significant. The history of previous thyroid surgery was significantly more frequent in group 1 than in group 2 (50% vs 13%; p=0.03). The CC length of the mediastinal thyroid gland was significantly longer in group 1 than in group 2 (77±11 mm vs 31±21 mm, respectively; p=0.0001). The volume of the mediastinal component was 264±106 cm^3^ and 40±41 cm^3^ in groups 1 and 2, respectively (p=0.0001) (Table 1).

**Figure 2:**
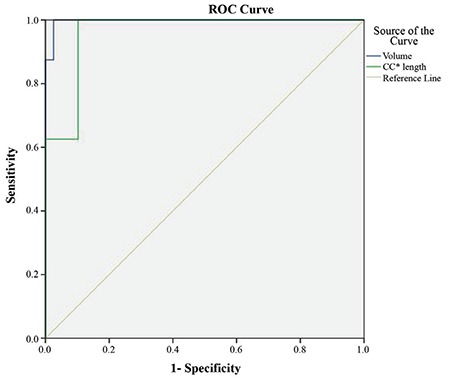
Receiver operating characteristic curve of the ability to predict an extra-cervical approach for craniocaudal length and volume of the mediastinal thyroid component of retrosternal goiter in the whole group of patients (Craniocaudal length; AUC: 0.962, standard error: 0.027) (volume; AUC: 0.997, standard error: 0.005).

The ROC curve analysis of CC length and the volume of the mediastinal component identified ≥66 mm [area under the curve (AUC): 0.962; standard error: 0.027] and ≥162 cm^3^ (AUC: 0.997; standard error: 0.005) as the cut-off values with the maximum accuracy, respectively (Figure 2). The CC length of the thyroid mass below the thoracic inlet ≥66 mm or a volume of the mediastinal portion ≥162 cm^3^ were significantly associated with an extra-cervical approach (p=0.0001) (Table 2). For predicting an extra-cervical approach, the sensitivity, specifity, positive predictive value (PPV), negative predictive value (NPV) and the accuracy of the cut-off value for CC length and mediastinal volume were indicated in Table 3.

### The impact of craniocaudal length and volume of the mediastinal thyroid component on the rate of the extra-cervical approach in patients without previous thyroid surgery

**Figure 3:**
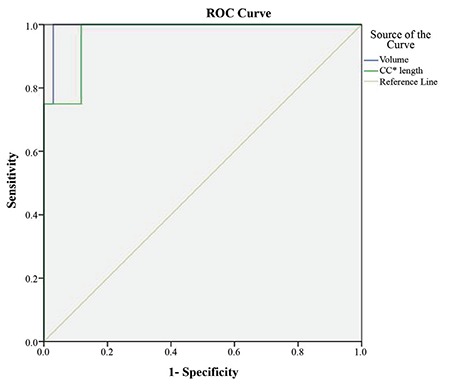
Receiver operating characteristic curve of the ability to predict an extra-cervical approach for craniocaudal length and volume of the mediastinal thyroid component of retrosternal goiter in the patients without previous thyroid surgery (Craniocaudal length; AUC: 0.971, standard error: 0.031) (volume; AUC: 0.993, standard error: 0.012).

Previous thyroid surgery was found to be a significant determining factor for extra-cervical approach in our patients. Therefore, we analyzed the impact of CC length and volume of the mediastinal thyroid component on the need for an extra-cervical approach in a subgroup of 38 patients excluding the 9 patients with previous thyroid surgery. An extra-cervical approach was necessary for 4 (10.5%) of the 38 patients. In the patients with extra-cervical approach, the CC length and volume of the mediastinal thyroid gland showed a significant difference compared to those with the cervical approach (75±6 mm vs 30±22 mm and 283±135 cm^3^ vs 35±38 cm^3^, respectively; p=0.0001). ROC curve analysis of CC length and the volume of the mediastinal component identified ≥69 mm (AUC: 0.971; standard error: 0.031) and ≥171 cm^3^ (AUC: 0.993; standard error: 0.012) as the cut-off values with the maximum accuracy, respectively (Figure 3). The rate of extra-cervical approach was 43% in patients with CC length of the thyroid mass below the thoracic inlet ≥69 mm whereas it was 3% in those with CC length <69 mm (p=0.002). Of the patients with a mediastinal thyroid volume ≥171 cm^3^, 80% underwent extra-cervical approach whereas none of the patients with thyroid volume <171cm^3^ needed an extra-cervical approach (p=0.0001) (Table 4). In patients with no previous thyroid surgery, the sensitivity, specifity, PPV, NPV and the accuracy of the cut-off value for CC length and mediastinal volume for predicting an extra-cervical approach were indicated in Table 5.

## DISCUSSION

The aim of the current study was to evaluate the impact of the volume of the mediastinal component of the thyroid gland on the necessity of using an extra-cervical approach in patients with RSG. We showed that a mediastinal thyroid volume ≥162 cm^3^ was a significant determining factor for an extra-cervical approach, with an NPV for the extra-cervical approach of 100% for RSG with smaller volumes.

Many definitions have been proposed to characterise RSG, and most of the definitions use the term RSG to refer to the partial or complete extension of the thyroid gland under the thoracic inlet or manubrium (12,15,16). In other definitions of RSG, certain anatomic points, such as the fourth thoracic vertebra, the aortic arch, and the level of the carina are identified; notably, the thyroid gland must extend to these anatomical points or must be substernal by at least 50%. In our study, RSG was defined as part of the thyroid gland being continually below the thoracic inlet based on physical examination or imaging.

The preferred treatment option for patients with RSG is surgery, even if they are asymptomatic, due to the potential risk of tracheal compression or malignancy (2,7,9,12,16,17,18). The preoperative identification of patients with RSG who will probably require the extra-cervical approach is important for the preoperative preparation of special equipment, the arrangement of optimal postoperative care conditions and to inform the patient about the morbidity associated with this procedure (12,15,18). For patients with RSG, CT is the most useful imaging modality for revealing the level of the mediastinal extension of the substernal thyroid gland and its association with the adjacent structures and vessels (10,11,14,15).

In the literature, numerous studies have suggested different classification systems for predicting the likelihood of using the extra-cervical approach in patients with RSG. Some investigators have identified that the rate of sternotomy was higher among patients with RSG whose goiter was below the lower limit of the aortic arch or extended toward the posterior mediastinum, or in the presence of ectopic mediastinal goiter, recurrent goiter or malignancy adhered to mediastinal structures (13,14,15,19). Huins et al. (15) conducted an a review encompassing 34 studies involving 2426 patients and recommended a simple classification system to predict the type of approach for RSG. The authors identified that cervical incision was enough for thyroidectomy for 84% of the 2426 patients; however, a sternotomy or thoracotomy was required for 16% of the patients (15). The investigators created a system comprising 3 grades according to the substernal extension of the thyroid gland being (1) above the aortic arch, (2) at the level between the aortic arch and pericardium and (3) extending below the level of the right atrium. In this analysis, it is emphasised that cervical incision is sufficient for patients whose thyroid gland is above the level of the aortic arch; however, partial or complete sternotomy is safer for those with thyroid glands at levels 2 or 3. Mercante et al. (14) suggested another classification system based on CT results. According to this system, the CC length in the CT images of RSG was divided into 3 grades, and the aortic arch was selected as the critical anatomical landmark. Extension from the thoracic inlet to the convex part of the aortic arch was classified as grade 1, while extension to the area between the convex and concave parts of the aortic arch or below the concave part of the aorta was classified as grade 2 or 3, respectively. The authors also defined 3 types with regard to the anteroposterior dimension: type A (prevascular), type B (retrovascular-paratracheal) and type C (retro tracheal). In their study, the rate of extra-cervical approach was 6.7%. An extra-cervical approach was significantly correlated with ≥ grade 2 RSG and/or retro tracheal extension and malignancy. Mercante et al. (14) found that the mean value for the CC length of the RSG was 11.08±3.32 cm in patients who received a sternotomy. However, the authors did not compare the CC length of the patients who underwent cervical incision or sternotomy. In our study, the CC length of the part of the thyroid gland under the thoracic inlet was 77±11 mm in patients who required an extra-cervical approach, and the cut-off value of CC length, which most accurately predicted an extra-cervical approach, was ≥66 mm. Although these values are smaller than those in the data of Mercante et al. (14), the cut-off value for CC length had an NPV of 97% in predicting an extra-cervical approach in our patients.

Raffaelli et al. (20) reported a low rate (0.6%) of extra-cervical approach in 355 patients treated surgically for RSG and identified the primary intrathoracic goiter or recurrent carcinoma as case-specific factors requiring an extra-cervical approach.

In many studies, various other risk factors that necessitate the extra-cervical approach were identified, such as airway emergency, superior vena cava syndrome, subcarinal extension and harder thyroid tissue (12,21).

Based on a general assessment of the findings in the literature, the extension of goiter below the level of the aortic arch or toward the posterior mediastinum seems to be the most important anatomical factor that influences the decision for using the extra-cervical approach. Two recent studies involving a large number of patients determined that extension below the aortic arch was the most prominent risk factor for a sternotomy, with an NPV of 97% for sternotomy for less extensive goiters (22,23). Other important factors include previous thyroid surgery, thyroid malignancy, primary mediastinal goiter, a mass heavier than 260 g and more than 70% of the thyroid gland being located in the mediastinum in the form of an iceberg (12). Mercante et al. (14) found the rate of sternotomy among patients with thyroid cancer to be 12 times higher than the rate for patients with benign pathology. In our patients, previous thyroid surgery was found to be a significant determining factor for the extra-cervical approach. In patients with RSG, initial mobilization of the upper thyroid pole helped deliver the retrosternal portion of the gland through the cervical incision by gentle traction over the upper thyroid pole (20). We preferred this surgical technique in all of our patients. However, in our patients with a previous cervical thyroidectomy, the absence of evident cervical thyroid tissue to apply gentle traction and presence of dense scar tissue obscured the removal of the retrosternal thyroid gland from the cervical incision. Although we found that the rates of hyperthyroidism and posterior mediastinal extension were higher in the extra-cervical approach group than in the cervical incision group, the difference between the 2 groups was not statistically significant, probably due to the small sample size of our study.

The correlation between the retrosternal extension level of RSG defined by CT findings and the need for the extra-cervical approach has been widely studied. However, we did not find any study in the literature that evaluated the correlation between the volume of RSG and the extra-cervical approach. To the best of our knowledge, our study is the first to evaluate the need for an extra-cervical approach in RSG according to the volume of the mediastinal component of the thyroid tissue. In our study, the cut-off value of the mediastinal thyroid volume, which predicted an extra-cervical approach with maximum accuracy, was ≥162 cm^3^. A mediastinal volume less than 162 cm^3^ was found to exclude the risk of using the extra-cervical approach with an NPV of 100% in our patients with RSG. In our study, in the subgroup of patients without a previous thyroid surgery, the cut-off value of mediastinal thyroid volume which predicted an extra-cervical approach was 171 cm^3^ and this cut-off value was also found to have an NPV of 100%.

The small patient population is a limitation of our study. Proper statistical analysis of independent variables, such as posterior extension, bilateral RSG, previous thyroid surgery and malignancy, could not be performed. Additionally, we were unable to conduct multivariate analysis including all of the independent variables that might have affected the risk of using the extra-cervical approach. However, analyzing the influence of these parameters on the rate of the use of the extra-cervical approach was not the primary aim of our study.

In conclusion, preoperative CT provides valuable information on RSG with respect to the extent of mediastinal involvement and the association of the thyroid gland with adjacent structures. The volume of the mediastinal component of the thyroid gland, which can be estimated using multi-sliced CT scan images, might be helpful to predict or exclude the necessity of using an extra-cervical approach with high sensitivity and NPV in patients with RSG. In our study, a thyroid volume of ≥162 cm^3^ extending below the thoracic inlet was a significant determining factor for an extra-cervical approach, with an NPV for the extra-cervical approach of 100% for RSG with smaller volumes. Further studies with an increased number of patients are needed to determine the value of volumetric analysis of the mediastinal portion of the thyroid gland in patients with RSG to predict the need for an extra-cervical approach.

## Figures and Tables

**Table 1:**
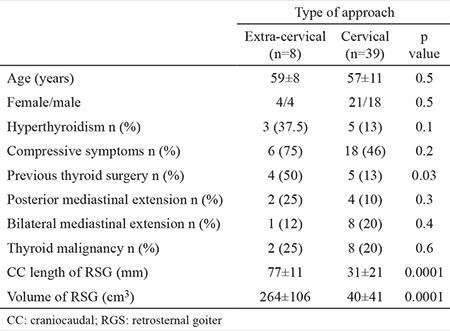
Demographic data, clinical features and computerised tomography findings in patients with a cervical and extra-cervical approach for retrosternal

**Table 2:**
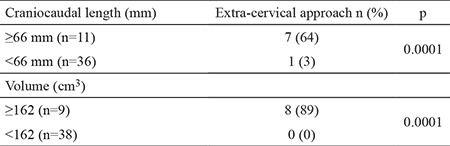
The rate of extra-cervical incision according to the cut-off values for craniocaudal length and volume of the mediastinal thyroid mass

**Table 3:**
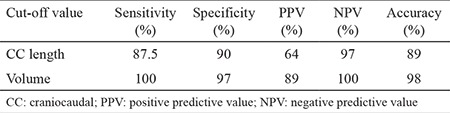
The sensitivity, specificity, positive predictive value, negative predictive value and the accuracy of the cut-off values of craniocaudal length and volume of the mediastinal thyroid mass for predicting an extra-cervical approach for retrosternal goiter

**Table 4:**
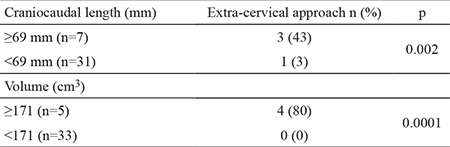
The rate of extra-cervical incision according to the cut-off values for craniocaudal length and volume of the mediastinal thyroid mass in retrosternal goiter patients without previous thyroid surgery

**Table 5:**
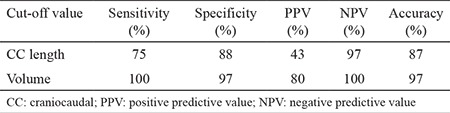
The sensitivity, specificity, positive predictive value, negative predictive value and the accuracy of the cut-off values of craniocaudal length and volume of the mediastinal thyroid mass for predicting an extra-cervical approach for retrosternal goiter in patients without previous thyroid surgery
